# Optimization of RNA Extraction from Rat Pancreatic Tissue

**Published:** 2014-05

**Authors:** Sanaz Dastgheib, Cambyz Irajie, Raheleh Assaei, Farhad Koohpeima, Pooneh Mokarram

**Affiliations:** 1 Department of Biochemistry, School of Medicine, Shiraz University of Medical Sciences, Shiraz, Iran;; 2 Student Research Committee, School of Medicine, Shiraz University of Medical Sciences, Shiraz, Iran;; 3 Department of Resource Development and Management, Shiraz University of Medical Sciences, Shiraz, Iran;; 4 Endocrinology and Metabolism Research Center, Nemazee Hospital, Shiraz University of Medical Sciences, Shiraz, Iran;; 5 Gasteroenterohepatology Research Center, Nemazee Hospital, School of Medicine, Shiraz University of Medical Sciences, Shiraz, Iran;; 6 Faculty for Advanced Biomedical Sciences, School of Medicine, Shiraz University of Medical Sciences, Shiraz, Iran

**Keywords:** Extraction, RNA, Pancreas, Autolysis

## Abstract

**Background:** Optimized RNA extraction from tissues and cell lines consists of four main stages regardless of the method of extraction: 1) homogenizing, 2) effective denaturation of proteins from RNA, 3) inactivation of ribonuclease, and 4) removal of any DNA, protein, and carbohydrate contamination. Isolation of undamaged intact RNA is challenging when the related tissue contains high levels of RNase. Various technical difficulties occur during extraction of RNA from pancreatic tissue due to spontaneous autolysis. Since standard routine protocols yield unacceptable results in pancrease, we have designed a simple method for RNA extraction by comparing different protocols.

**Methods: **We obtained 20-30 mg pancreatic tissues in less than 2 min from 30 rats. Several methods were performed to extract RNA from pancreatic tissue and evaluate its integrity. All methods were performed three times to obtain reproducible results.

**Results: **Immersing pancreatic tissue in RNA-later for 24 h at -80ºC yielded high quality RNA by using the TriPure reagent which was comparable to the commercial RNeasy Micro Kit. The quality of RNA was evaluated by spectrophotometer, electrophoresis and RT-PCR. We separated intact 28S and 18S ribosomal RNA (rRNA) when our procedure was compared with the RNeasy Micro Kit. Finally, full length of the actin gene was amplified by RT-PCR.

**Conclusion: **We designed a simple, fast, cost-effective method for complete RNA extraction from the least amount of quantitatively intact pancreatic tissue.

## Introduction

Information of a structural gene is usually transcripted to a functional product by gene expression. Recent studies have focused on RNA analysis as a gene expression tool in cells to detect differential gene expression between two conditions. Different methods have been presented for extracting nucleic acids such as guanidinium thiocyanate followed by phenol-chloroform extraction, chromatography by cellulose, extraction using silica matrices, magnetic bead based nucleic acid purification, and anion-exchange.^1^^,^^2^

Accurate detection of gene expression is influenced by status of the RNA that is isolated from tissues. The quality of isolated RNA should be checked prior to its use in subsequent tests and studies. The purity and quality of the isolated RNA is a vital step in RNA dependent assays. Performing complementary molecular tests with low-quality RNA may compromise the results of downstream applications which are often labor-intensive, time consuming, and highly expensive. Researchers need high quality RNA for molecular biological tests that have various diagnostic applications such as quantitative RT-PCR, micro-arrays, ribonuclease protection assay, northern blot analysis, RNA mapping, and cDNA library construction.^3^^,^^4^

The quality of purified RNA from tissues and cells is variable. Often, after extraction, RNA is rather unstable over a long storage time. Long mRNA fragments up to 10 kb are especially sensitive to degradation.^5^^,^^6^

Researchers must consider various factors that affect the quality of purified RNA. Purified RNA must not be contaminated with RNases, proteins, genomic DNA, and enzymatic inhibitors. Additionally, the UV absorption ratio (260/280) of total RNA should be between 1.8-2.0 and RNA should have a minimal degree of fragmentation during electrophoresis. Recently developed laboratory techniques allow scientists to more adequately control the quality of samples used for molecular analyses.^7^^,^^8^

Although RNA is easily and successfully isolated from most cells and tissues, intact RNA extraction from the pancreas is difficult due to the high level of its ribonucleases (RNases). Despite the improvement in several approaches, including rapid removal of pancreatic tissue from the abdominal cavity and homogenization at cold temperatures to inhibit RNases, the isolation of intact, high-quality RNA from this tissue remains challenging because of the complexity and indefinite reproducibility of the above mentioned techniques.^9^^-^^15^

We aimed to design a simple, fast, and cost-effective method for complete RNA extraction that utilized the least amount of pancreatic tissue. We compared different protocols of RNA extraction and optimized the most feasible extraction method by which the highest quality RNA could be qualitatively obtained. 

## Materials and Methods

In the current study, pancreatic tissues were taken from 30 rats and divided into several pieces (20-30 mg) for use in the following methods.

**Figure 1:**
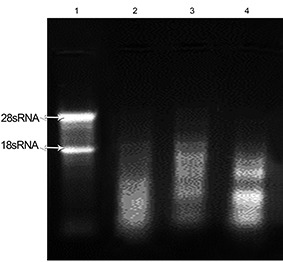
Evaluation of total RNA integrity isolated from three snap-frozen pancreatic tissues using RNX-plus. Lane 1 shows the quality of RNA extracted from the liver as the control. Lanes 2-4 represent the quality of 28S/18S rRNA bands in total RNA extracted from three pancreatic tissues.

**Figure 2:**
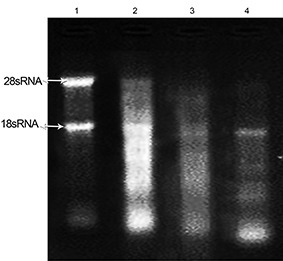
Evaluation of total RNA integrity isolated from three snap-frozen pancreatic tissues using TriPure solution. Lane 1 shows the quality of RNA extracted from liver tissue as the control. Lanes 2-4 represent the quality of 28S/18S rRNA bands in total RNA extracted from three pancreatic tissues.

In the first method, these small pieces of pancreatic tissue from 30 rats were placed into two microtubes. The first tube contained 1 ml RNX-plus solution (Cinnagen, Tehran, Iran) and the second tube contained 1 ml TriPure isolation reagent (Roche Applied Science, Germany). Both solutions contained guanidinium thiocyanate which inhibits RNase. Subsequently, both tubes were snap-frozen in liquid-nitrogen for inhibition of RNase activity after which the integrity of RNA was evaluated with denaturing agarose gel electrophoresis (figures 1 and 2).

In the second method, pancreatic tissues were perfused with 1 ml RNA-later as the RNA stabilization reagent (Qiagen, USA) by an insulin syringe. Tissues were subsequently cut into small pieces with sterile scissors. The tubes that contained pancreatic tissue and RNA-later were processed for extraction by using the RNX-plus solution, TriPure, and RNeasy Micro Kits (Qiagen, USA) according to the manufacturers’ instructions after either 30 min, overnight in 4ºC, or following storage at -80ºC for one, three or seven days in order to compare the effect of preservation time on RNA integrity. In all conditions, the livers were removed from 30 rats and used as control tissue in a comparison of RNA quality between pancreatic and liver RNAs. 

The isolated RNAs were separately diluted at a ratio of 1/100 in water treated with diethylpyrocarbonate (DEPC) and the 260/280 UV absorption ratio was calculated. The integrity of total RNAs was evaluated by denaturing agarose gel (MOPS gel) electrophoresis. MOPS buffer was used as running buffer to separate several ribosomal RNA (rRNA) bands (28S, 18S, and 5S) during electrophoresis.^16^

## Results

We did not obtain acceptable bands when RNA was extracted with the RNX-plus reagent or RNA-later. However, we observed the best results when TriPure reagent was used. These results were dependent upon the tissue preservation time, temperature and perfusion method. Immersion of pancreatic tissue in RNA-later for 24 h at -80ºC yielded high quality RNA with sharp, distinct 28S/18S bands.

*Evaluating RNA Integrity with the RNX-Plus Solution*

No specific band was seen when we used the RNX-plus solution. According to electrophoresis results, the RNA was completely degraded (figure 1).

*RNA Integrity with TriPure Reagent*

In comparison to the liver tissue control, we noted that RNA separation was not successful when the TriPure reagent was used (figure 2).

*RNA Integrity of Samples Immersed in RNA-Later and Extracted with RNX-Plus or TriPure Reagent*

**Figure 3:**
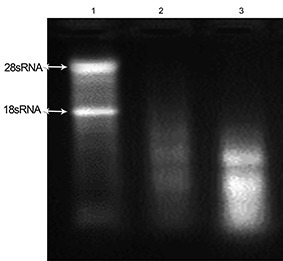
Electrophoresis and RNA integrity analysis of total RNA isolated from two snap-frozen pancreatic tissues by using RNA-later and RNX-plus reagent. We immersed tissues in RNA-later after which they were snap-frozen in liquid nitrogen, followed by RNA extraction with RNX-plus reagent. Lane 1 shows the quality of RNA extracted from liver tissue as the control. Lanes 2, 3 represent the quality of 28S/18S rRNA bands in total RNA extracted from two pancreatic tissues.

**Figure 4:**
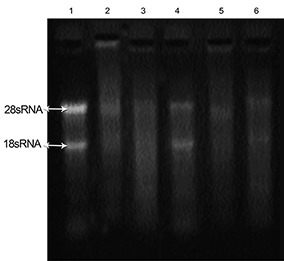
Electrophoresis and RNA integrity analysis of total RNA isolated from snap-frozen pancreatic tissues by immersing samples in RNA-later and TriPure reagent. We immersed the tissues in RNA-later after which they were snap-frozen in liquid nitrogen, followed by RNA extraction with TriPure reagent. Lane 1 shows the quality of RNA extracted from liver tissue as the control. Lane 2 shows the status of RNA extracted from pancreatic tissue immediately after immersing in RNA-later. Lane 3 shows the status of RNA extracted after 24 h at 4ºC, lane 4 shows the status of RNA extracted after 24 h at -80ºC, lane 5 shows the status of RNA extracted after 3 days at -80ºC, and lane 6 represents the status of RNA extracted after 7 days at -80ºC.

**Figure 5:**
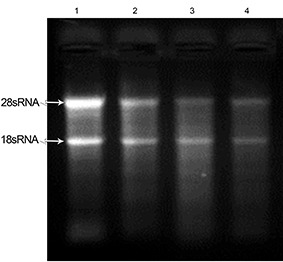
Evaluation of total RNA integrity in snap-frozen pancreatic tissues immersed in RNA-later for 24 h at -80ºC which were isolated with TriPure reagent. Lane 1 shows the status of RNA extracted from liver tissue as the control, lanes 2-4 show the status of RNA extracted from three rats after tissues were immersed in RNA-later, then immediately placed in liquid nitrogen and preserved for 24 h at -80ºC, followed by extraction with TriPure reagent. Lanes 2-4 represent the reproducibility of the RNA extraction by TriPure when tissues that contained RNA-later were stored for 24 h at -80ºC.

There was no band visualized when we used RNA-later along with the RNX-plus reagent (figure 3). Depending on the duration of preservation and temperature, the TriPure reagent was able to produce RNAs with different integrities (figures 4 and 5). However the only considerable band (28S/18S rRNA) was seen when pancreatic tissues were immersed in RNA-later for 24 h at -80ºC.

*RNA Integrity with RNA-Later and the Qiagen Kit*

**Figure 6:**
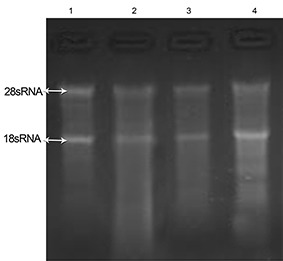
Evaluation of total RNA integrity in snap-frozen pancreatic tissues immersed in RNA-later for 24 h at -80ºC that were isolated with the Qiagen kit. Lane 1 shows the status of RNA extracted from rat liver tissue as the control using the same protocol. Lanes 2-4 show the status of RNA extracted after tissues were immersed in RNA-later and immediately placed in liquid nitrogen for 24 h at -80ºC, followed by RNA extraction with Qiagen RNeasy Micro Kits.

In terms of purity and integrity, high-quality RNA was extracted by using RNA-later along with the Qiagen reagent (figure 6).

*RT-PCR for β-Actin Gene Expression in Pancreatic Tissues with RNAs Extracted by Using the TriPure Reagent*

**Figure 7:**
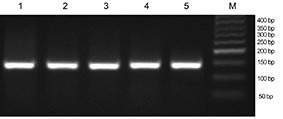
RT-PCR analysis of mRNA isolated from snap-frozen pancreatic tissues immersed in RNA-later for 24 h at -80ºC that were extracted with TriPure reagent. Lane M represents 50 bp DNA markers and lanes 1-5 show β-actin (138 bp) cDNAs amplified by RT-PCR.

High quality RNA extracted with the TriPure procedure amplified a specific β-actin locus. The full length of actin was amplified by RT-PCR in order to evaluate the synthesized cDNA by intact RNA. The presence of 138 bp bands confirmed the cDNA quality (figure 7).

## Discussion

Obtaining high-quality RNA is the first and most critical step in many molecular techniques such as reverse transcription real-time PCR (RT-qPCR), transcriptome analysis that uses next-generation sequencing, array analysis, northern analysis, and cDNA library construction. RNA extraction is complicated because of the presence of ribonuclease enzymes in cells and tissues that can rapidly degrade RNA.^14^^,^^17^^,^^18^

RNases act without cofactors and are stable enzymes. The inactivation of RNases is difficult. Small amounts of RNase are sufficient to destroy RNA. In order to inactivate any RNases prior to surgery and in cases of removal of rat pancreatic tissue from the abdominal cavity, all surgical instruments should be cleaned with strong detergents, thoroughly rinsed, and placed in an oven for at least 4 h at 240ºC. The place of surgery should be sterilized with NaOH and mild bleach to inactive the RNases.^19^

The pancreas has an extremely high level of RNase. RNA degradation occurs while the pancreas tissue is removed during dissection. In order to increase efficiency dissection should be performed as quickly as possible.^18^^,^^19^

The pancreas is one of the important tissues that functions in the body’s homeostatic mechanisms. Therefore, improvement of RNA extraction procedures from the pancreas increases understanding of active pathways such as glucose regulation and redifferentiated β islets of the pancreas before and after treatment with different drugs. Knowledge of gene expression assays may potentially lead to the development of therapeutic drugs to restore β-cells and prevent apoptosis in diabetics.^20^

To identify novel metabolic genes and pathways that may play a role in the pathogenesis or treatment of diabetes, differential expression of metabolic genes is necessary. However, RNA extraction from pancreatic tissue is difficult because of the abundance of RNase.^21^^-^^23^ Therefore we have attempted to design an efficient, simple optimized method to extract RNA from the pancreas based on our laboratory facilities.

In order to enhance and correct this method, we assessed different common RNA extraction methods from pancreatic tissue with particular focus on the effect of frozen storage and RNase inhibition strategies, both of which affect RNA quality.

The duration of surgery and amount of collected pancreatic tissue are the most crucial steps for obtaining intact RNA. Recent studies have shown a positive correlation between RNA degradation and the amount of pancreatic tissue.^10^^,^^15^^,^^18^ Therefore, in this study we obtained only 20-30 mg of pancreatic tissue in less than 2 min from the anesthetized rats.

Several methods are used in molecular biology to isolate RNA from different samples. The most common isolation method is guanidinium thiocyanate followed by phenol-chloroform extraction that uses liquid nitrogen along with a specialized motorized grinding device which prevent RNA activity.^24^ RNA extraction performed with guanidinium thiocyanate followed by phenol-chloroform on different tissues has yielded good results, with the exception of pancreatic tissue (data not shown). Several studies determined that the RNA isolation procedure must include a number of important steps before, during, and after the actual RNA purification.^25^ Therefore we changed the RNA extraction process by using RNA-later.

Our results showed that RNA later rapidly permeated the pancreatic tissues, protected cellular RNA and minimized the need to immediately process the tissue samples. The best results were obtained after the samples that contained RNA-later were stored for 24 h at -80ºC. Our data confirmed that it was necessary to stabilize RNA within the pancreatic samples by using an effective reagent to delay RNA degradation, even the extraction was performed by using a Qiagen RNA extraction kit. 

Several kits such as TriPure and Qiagen (foreign kits) and RNX-Plus (homemade, Iran) are commercially available for RNA extraction in Iran. These kits offer the dual advantages of ease of use and effectiveness. These kits often work well and are widely used. Foreign kits are also more expensive per sample than homemade kits. 

We were unable to obtain high-quality RNA with Iranian reagents and snap-frozen tissues. Possibly, the low yield of RNA from the immediately frozen pancreatic samples was attributed to rapid degradation initiated by RNases in the pancreas. Although our entire experimental process was similar to a previous study that used TRIzol reagent where the researchers obtained high-quality intact RNA from the rat pancreas,^15^ however we were unable to obtain good quality RNA. The only difference between these two protocols was the use of RNX-plus in our study which did not seem to be an appropriate solution for RNA extraction from pancreatic tissues compared to TRIzol. Although RNX-plus works well for extracting RNA from other tissues, we did not use RNX-plus for RNA extraction from pancreatic tissue. In this case, the possibility of the presence of active RNase during surgery possibly led to RNA degradation and could not be ruled out.

In order to evaluate the quality of RNX-plus, the second RNA extraction procedure was performed using the TriPure reagent under snap-frozen conditions which led to decreased RNA degradation. However, the problem with using TriPure solution was the lack of reproducibility. In the second step, we decided to decrease autolysis during dissection and RNA extraction by using RNA-later as a pancreas RNase inhibitor. 

The use of RNA-later before starting the isolation method with RNX-plus did not change the quality of RNA. The RNA was degraded and 28S/18S rRNA smear bands were observed. However, when TriPure was used, RNA quality was incredibly high. Additionally, this process was reproducible. The quality of RNX-plus was questioned. We also detected DNA contamination with the use of RNX-plus that had to be reduced.

In the third step we have focused on how to perfuse RNA-later into the pancreatic tissues. Complete tissue perfusion with RNA-later after total pancreatic tissue dissection is not cost-effective. Therefore, we researched three perfusion conditions for the later use of RNA as follows. We dissected a small section of the pancreas (20-30 mg) during surgery from anesthetized rats and immersed these tissues in 1 ml RNA-later for 30 min at 4^°^C or for one, three and seven days at -80°C. As shown in figure 4, the optimum time for the best results was storage for 24 h.

The above mentioned methods enabled a smaller amount of RNA-later to penetrate into the organ. The degradation process was halted faster because small pieces were dissected. 

Modifications to the basic procedures introduced by Li and Griffin et al enabled us to obtain high-quality reproducible RNA from rat pancreatic RNA which was suitable for RT-PCR of the actin gene as shown in figure 7.

## Conclusion

Although isolation of intact RNA from the rat pancreas is compromised by autolysis and by the presence of endogenous RNases, our pancreas perfusion method yields excellent, high quality and integrity RNA for molecular biology studies which is comparable with Qiagen kits. In summary, the presented method is a simple, reproducible and economical procedure which does not require the use of higher amounts of RNA-later total perfusion. Using TriPure solution after RNA-later perfusion can be a good substitute for expensive and column-based RNA extraction kits. Furthermore, use of the RNX-plus kit for RNA extraction from pancreatic tissue is not recommended. 
